# The Evolution of Technology and Physical Inactivity: The Good, the Bad, and the Way Forward

**DOI:** 10.3389/fpubh.2021.655491

**Published:** 2021-05-28

**Authors:** Mary N. Woessner, Alexander Tacey, Ariella Levinger-Limor, Alexandra G. Parker, Pazit Levinger, Itamar Levinger

**Affiliations:** ^1^Institute for Health and Sport (iHeS), Victoria University, Melbourne, VIC, Australia; ^2^Australian Institute for Musculoskeletal Science (AIMSS), University of Melbourne and Western Health, St Albans, VIC, Australia; ^3^Independent Researcher, Tel Aviv, Israel; ^4^Centre for Youth Mental Health, University of Melbourne, Melbourne, VIC, Australia; ^5^National Ageing Research Institute, Melbourne, VIC, Australia; ^6^Rehabilitation, Ageing and Independent Living Research Centre, Monash University, Melbourne, VIC, Australia

**Keywords:** physical activity, technology, behaviour change, obesity, inactivity

## Abstract

Since the beginning of time people explored and developed new technologies to make their activities of daily living less labour intense, more efficient and, consequently, more sedentary. In addition, technological advances in medicine throughout history have led to a substantial increase in life expectancy. However, the combination of increased sedentary behaviour and increased life-expectancy resulted in a sharp increase in overweight and obesity related chronic conditions and illness. Although people may live longer, they are doing so with poorer physical function and a reduced quality of life. In this review we explore how technological advances have influenced people's sedentary behaviour and, through the lens of the affective-reflective theory (ART), we propose a means by which technology could be repurposed to encourage greater engagement in physical activity.

## Introduction-Development of Technologies—The Good

Since the appearance of the species that walked upright on two legs, new technologies were developed to make life on this planet easier and more efficient. The homo habilis, who lived around 2.3 million years ago, were the first to develop tools from stones and are considered as the “handy man” of the species of the genus homo (1). The Homo erectus who lived around two million years ago and had a larger brain than the homo habilis, was the first to leave Africa, and the first to use fire for protection and cooking (2). Human history is full of technological advances in all facets of life.

**Figure 1:**
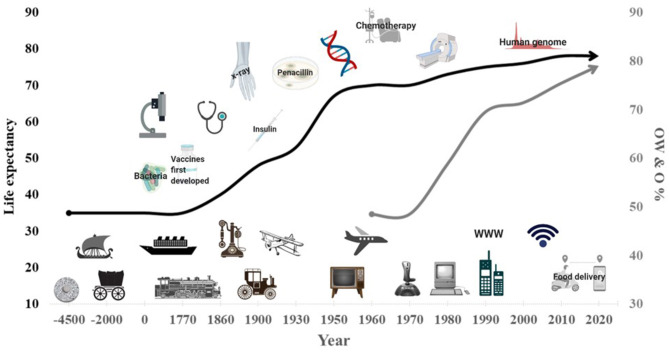
Increase in life expectancy (black line) and in overweight and obesity trends (OW and O, grey line) with advanced in technology and medical treatments. Created with BioRender.com.

Human inventions that were directed to make work and life less physically demanding started around six to seven thousand years ago with the invention of the wheel and the horse or cattle drawn cart. These inventions not only saved people the “effort” of walking, carrying and lifting, but opened a new era of trade and inspired the development of novel transportation modalities. These new ideas materialised during the industrial revolution (1750–1914) which led to the development of new transportation options on land, in the sea and in the air (Figure 1). Advanced technologies in business improved work efficiency and profit while reducing the amount of physical work needed, but during the second half of the twentieth century, and particularly after the 70s, began the “electronics and telecommunications revolution.” This time-period saw the widespread use of a variety of household appliances designed to improve communication (telephones) and decrease manual labour (computers, washing machines, vacuum cleaners) (Figure 1) (3).

The technological revolution was not limited to improving productivity, lifestyle, and leisure but was also critical to launching our modern medical field which started more than 2,000 years ago. Hippocrates, who is considered by many as the “father of medicine,” was the first to map the human anatomy and characterise diseases. He was also the first to treat diseases by focusing on changes to people's diet and physical activity, which in today's world we refer to it as “Exercise is Medicine” and/or “lifestyle medicine” (4). A breakthrough in medicine was the development of the first vaccine, for smallpox, around 1,770, with subsequent vaccines ultimately developed for anthrax, fowl cholera, tetanus, and others. The twentieth century was full of medical inventions that improved the early diagnosis of disease as well as treatment and management options. As a result, life expectancy, which was stable at 35–40 years of age for thousands of years, increased dramatically in the twentieth century and currently it is around 80 years (Figure 1).

## Sedentary Behaviour—The Bad

While advances in technology have provided many benefits to society, new technology has also led to a substantial reduction in the amount of incidental physical activity. Physical activities, previously conducted as part of a “standard” working day (active transport, labour, etc.) or as part of domestic duties around the home (cleaning and cooking), have been reduced or replaced by machines. The relatively recent development of the internet and its accessibility on mobile devices (phones, tablets, and others) has also negatively impacted our physical activity. There are established associations between internet usage during leisure time and sedentary behaviour and obesity in children and adults (5, 6). Indeed, the overall reduction in physical activity, irrespective of the cause, as well as the increase in the prevalence of sedentary behaviours are strongly associated with the development of one of the most serious health epidemics people have faced, the obesity epidemic, which can also be described as a syndemic as the risk of obesity is higher in those from low-socioeconomic status (SES) and pre-existing inequities and social determinants of health (7, 8). In the US, the prevalence of overweight and obesity “jumped” from <50% in the 1960s to almost 80% at present (9, 10). The health consequences of obesity are profound and include the onset of diabetes, hypertension and cardiovascular disease, mental and psychological conditions, and other chronic diseases. Physical inactivity is both a precursor and a consequence of obesity, and, importantly, middle-aged and older individuals who are physically inactive are at a high risk of muscle mass and strength loss, sarcopenia, falls, fractures, cognitive decline, and hospitalizations (11–15). Although people may be living longer, those later years before death, are often lived with disability or chronic disease that impact their functional capacity, independence, and quality of life (16). This concept of improvement in health and well-being inspired the United Nation in the Sustainable Development Goals (SDG) #3 to ensure health and well-being for all (17). As attributed to Abraham Lincoln, “And in the end it's not the years in your life that count; it's the life in your years.”

## The Benefit in Increasing Physical Activity and Reducing Sedentary Behaviour

There are extensive short term and long-term functional, physical, cognitive, clinical, and mental health benefits associated with maintaining a physically active lifestyle and reducing sedentary behaviours (18–23). Increased physical activity and exercise capacity is the corner stone of every lifestyle intervention for healthy and clinical populations at any age group due to the vast evidence for its effectiveness. Since the first physical activity guidelines and recommendations publications, over 40 years ago, by the American College of Sports Medicine, hundreds of exercise guidelines, for almost every single population were written by the leading exercise and clinical institutions in the world. Moreover, national and international guidelines and recommendations for minimum levels of physical activity have been established in both the government and health sectors of numerous nations (19, 24). Despite minor differences in specificities, most guidelines agree that 150 min a week is the threshold at which there are benefits to health (25). Meeting these guidelines results in about 75% of the total possible risk reduction for all-cause mortality and 50% reduction in cardiovascular disease mortality.

## Technologies and Active Lifestyle—The Way Forward

Despite the wealth of evidence demonstrating the benefits of physical activity, recent federal monitoring data suggest that as of 2015/2016 <30% of US adults and 20% of adolescents are meeting their respective physical activity guidelines for aerobic and strength exercises (26). A variety of factors contribute to physical inactivity including demographics, physical or mental health conditions, social and relational characteristics as well as environmental factors (27). The complex associations between intra and inter-individual factors as well as societal and economic factors that influence health behaviours create challenges in conceptualising and implementing successful interventions to promote behaviour change. Thus, while it was originally assumed that evidence based guidelines could encourage individuals to become more physically active, it is now quite apparent that this type of rational-educational messaging of needing to “move more” is not driving the population-level behaviour change that is required to address physical inactivity's contribution to the burden of disease (28, 29). Many theories have been formulated to address the issue of how to motivate people to change their behaviour and engage in sustained, long-term physical activity. In relation to our proposal of the interdependency of technology as a preferred choice of leisure-time activity and its relationships with physical inactivity trends, frameworks which link enjoyment of activities with the motivation and behaviour to habitual activity are most relevant to explore in this context.

The Affective-Reflective Theory (ART) of physical inactivity and exercise is a novel approach to explaining why individuals who are physically inactive do or do not commence and maintain physical activity (30). Unlike previous theories which purport that knowledge, planning and intrinsic value can drive motivation and behaviour change, ART suggests that a more immediate emotive association with physical activity could be a critical driver (30, 31). Underpinning ART is the belief that automatic affective associations of exercise (the immediate positive or negative responses a person has) initiate either an approach-oriented or an avoidance-oriented response to exercise. The ultimate response of either initiating exercise or remaining sedentary depends on the relative weight of both the perceived enjoyment of the new behaviour (exercise) vs. the affect associated with the current state (inactivity). An additional contributing element is the controlled reflective evaluation (e.g., an individual reflecting on their needs and values) (32). Individuals essentially need to have both of these aspects aligning in order to initiate an activity. The significant increases in internet accessibility through technological interfaces, that promote leisure time inactivity, have now been inextricably linked to associations with enjoyment and fulfilment which the idea of exercise (despite the knowledge of its benefits) cannot supersede.

One of the challenges in surpassing the enjoyment of the internet is that the websites and applications themselves are designed to encourage users to continually engage through individually designed algorithms (suggested content), as well as rewards and incentives (“likes,” notifications) (33). These specially designed user-experiences are uniquely effective due to this feedback triggering the release of dopamine, a neurotransmitter linked to reward-related learning, pleasure and, significantly, addiction (33, 34). With the frequency and duration of internet use increasing significantly over the last decade especially in the younger population (concomitant to a significant rise in internet addiction), promoting behaviour change that requires reduced use, or disuse, of these technologies could be even more challenging (35).

This presents the question of whether it is possible for the technological advances that contributed to reductions in physical activity to be repurposed to promote it. ART indicates that to create behaviour change the individual needs to understand the benefits of physical activity, they must value their own health and the behaviours that support it, and their immediate affective (emotional) response to the stimulus (activity) must create more perceived enjoyment than the alternative (inactivity) (30). By retooling the technology that brings enjoyment can we ultimately use technologies to promote and motivate engagement in long-term physical activity? This is critically important as is one of our greatest challenges from a Planetary Health perspective is searching for ways to incentivise healthy behaviour change (36).

## Emerging Evidence for the Use of Technology to Promote Physical Activity

The idea of using technology to encourage physical activity has been around since the emergence of personal electronic devices. The earliest iteration could arguably be the pedometer. These devices have transitioned to wearable activity trackers with the emergence of fitness bands, smartwatches, and accessories that track steps, physical activity, heart rate, and additional health-related data (37). A large meta-analysis found that interventions that provided physical activity trackers for older people improved physical activity and mobility, but not necessarily quality of life (38). Overall, the short term use of wearable activity trackers appears effective at increasing physical activity (steps per day) and reducing BMI, but the long-term effects on behaviour change have not been rigorously explored (20).

A recent qualitative study found that sustained use of activity trackers was influenced by an individual's perceived future value of data accumulation, opportunistic engagement and the feeling of empowerment, but that positive changes in behaviour were also inextricably linked to the individual's ability to self-set goals prior to usage (37). These findings and those of Ryan and Deci support the ART framework, suggesting that enjoyment and opportunistic engagement can create a positive affect with exercise but that the reflective evaluation of individual's goals and the intrinsic value of the activity remain critical (32).

**Figure 2:**
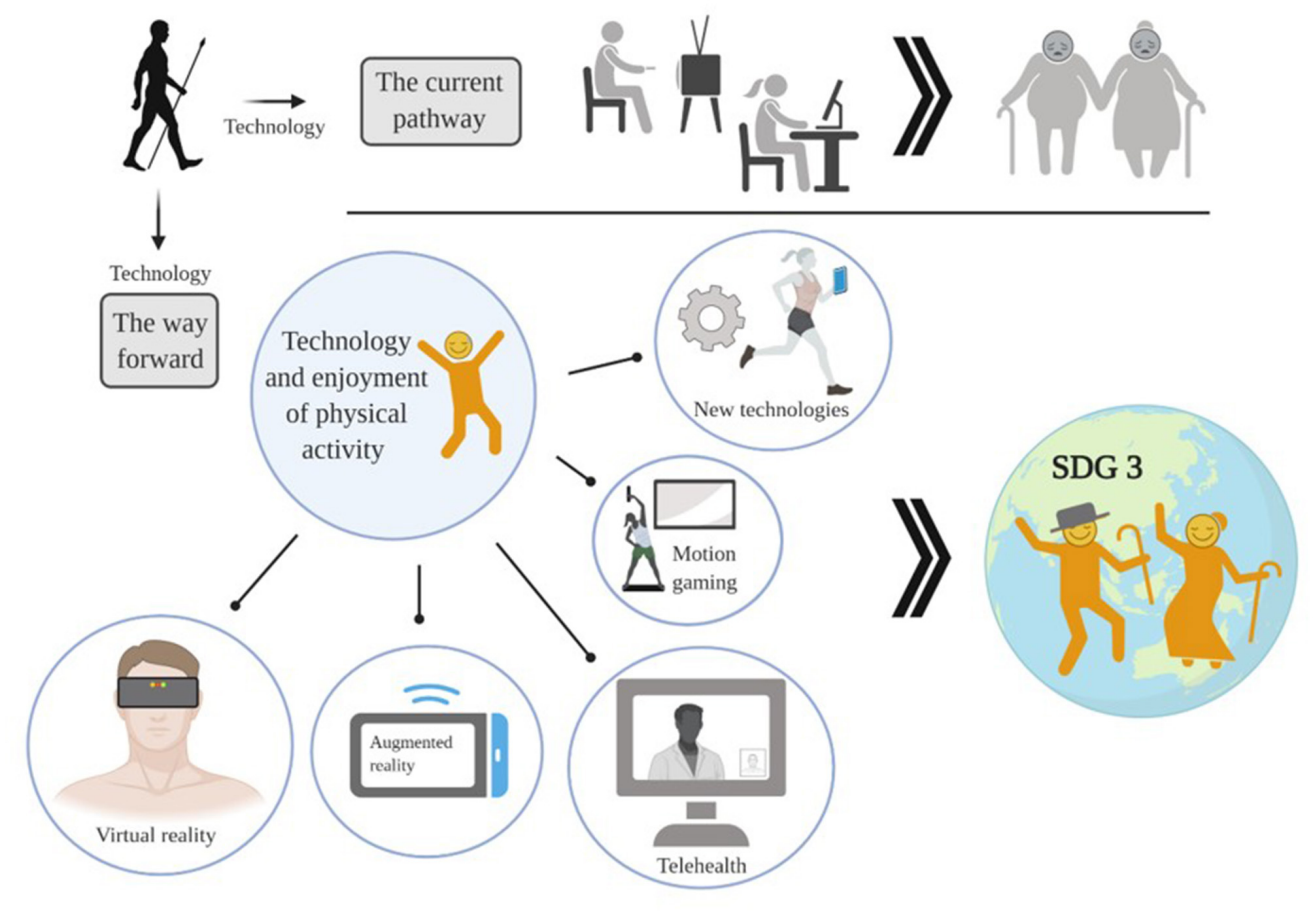
Currently technologies promote sedentary behaviour and physical inactivity, however, technologies have the vast potential to be repurposed to promote increased engagement and enjoyment of physical activity. This will contribute to the UN SDG#3 targets and indicators, ensuring health and well-being for all. Created with BioRender.com.

Recent advances in “exergaming” and virtual reality technologies also present an intriguing opportunity to reimagine how to engage in physical activity in the modern era. Given our premise that the enjoyment gleaned from gaming, social media, and other online interfaces is promoting physical inactivity, incorporating activity within these platforms is a promising approach for changing individuals' automatic decision-making processes and affective responses to physical activity. While the field as a whole is in its infancy, initial studies show some positive trends. Gamifying exercise through active video games can lead to reductions in cholesterol and body fat while increasing enjoyment and self-efficacy (39, 40). One study in healthy adults indicated that the improvements in blood glucose management from supervised exercise games were significantly greater than those adults completing standard exercise alone (40). With the recent emergence of virtual reality technology, the opportunities for novel means of gamifying exercise are endless. While the field is in its infancy, early studies suggest that exercising with virtual reality technology has the potential to improve physical and psychological well-being in a range of individuals (41). The accumulating evidence suggests some practical implications that should be explored. Increasing enjoyment of exercise through technology can lead to greater adoption of physical activity and better health outcomes and improved well-being, that in turn will assist in promoting the UN SDG #3. Perhaps rather than, or in addition to, focusing on decreasing screen time we need to explore the technologies that encourage or require movement while on these platforms (Figure 2).

The recent emergence of augmented and virtual reality technology in our mobile phones and headsets demonstrates how this technology-based physical activity could work in principle. There are a variety of online applications which utilise augmented reality or virtual reality to increase engagement in and enjoyment of physical activity through gamifying exercise (42, 43). These initial studies were relatively short in duration, and it is unknown whether these types of applications can be modified to induce long term behaviour changes that will result in clinically meaningful population-level improvements in obesity rates and physical function. Initial evidence also suggests that some of those embedded engagement prompts currently used to increase engagement in screen time (reminders, alerts, etc.) can also be used to prompt physical activity behaviours (44). Retooling current technology to promote movement and decrease sedentary behaviour would require a major industry shift in the success indicators for technology uptake. Currently they rely on purely quantitative measures of engagement (duration, frequency), whereas herein we propose the need for a focus on social and public health benefits. Embedding public health researchers and discipline experts within the technology teams can help ensure there is both an economic and a health benefit within the products.

## Discussion

The historical evolution of mankind has become inextricably linked with the development of technology. Early advances in technology improved both our lives and livelihoods, but with unforeseen consequences to our health and well-being in terms of obesity and physical inactivity. Our over-reliance on technology has transformed what used to be tools to assist in labour and leisure into personal devices we now rely on for enjoyment.

Uptake of and adherence to physical activity remains a critical challenge for health promotion, with a key barrier being a lack of enjoyment of the physical activity. Improving individuals' relationship with physical activity by creating positive associations and maximising enjoyment should be a key focus of future research. Our current utilisation of technology for leisure reinforces and promotes the very physical inactivity we are trying to reduce. However, the emergence and increasing popularity of either gamifying exercise or embedding exercise within the technology presents a viable target for innovative solutions to urgent public health concerns.

Short term studies have indicated strong efficacy and enjoyment of a variety of technology platforms (accelerometers, phone applications, online interventions) targeting increases in physical activity and improvements in a variety of health parameters (38, 45, 46). Phone-based applications and physical activity trackers hold particular promise due to their accessibility and widespread utilisation. If the use of these technologies can be promoted within clinical practise as a component of health behaviour modification the impact could be profound (47). In tandem with this avenue, collaborations between the technology and health industries are essential to ensure the advances in technology support positive health behaviours.

## Conclusion

The current trajectory of the physical inactivity trend and its link with the advances in technology are concerning. Technology is now an inextricable part of almost every aspect of our lives, yet it does not always improve our quality of life. But with continuous advances in technology, as well as our understanding of the principles underlying human behaviour change, comes a unique opportunity to reimagine what the nexus between physical activity and technology could look like in the twenty-first century, and beyond.

## Author Contributions

MW and IL drafted the manuscript. AT, AL-L, AP, and PL provided critical review of the manuscript. All authors reviewed and approved the final manuscript.

## Conflict of Interest

The authors declare that the research was conducted in the absence of any commercial or financial relationships that could be construed as a potential conflict of interest.
